# Using opportunistic sightings to infer differential spatio-temporal use of western Mediterranean waters by the fin whale

**DOI:** 10.7717/peerj.6673

**Published:** 2019-03-29

**Authors:** Estefanía Torreblanca, Juan Antonio Camiñas, David Macías, Salvador García-Barcelona, Raimundo Real, José Carlos Báez

**Affiliations:** 1 Departamento de Biología Animal, Universidad de Málaga, Málaga, Spain; 2 Centro Oceanográfico de Málaga, Instituto Español de Oceanografía, Fuengirola, Spain; 3 Centro Oceanográfico de Canarias, Instituto Español de Oceanografía, Santa Cruz de Tenerife, Spain; 4 Facultad de Ciencias de la Salud, Universidad Autónoma de Chile, Santiago de Chile, Chile

**Keywords:** *Balaenoptera physalus*, Cetaceans, Spatio-temporal modeling, Gulf of Lion, Balaenopteridae, Habitat use

## Abstract

The fin whale (*Balaenoptera physalus*) is a cosmopolitan species with a resident population in the Mediterranean Sea. Due to its habitat, open seas often far from ports and airfields, and its long-distance migratory behaviour, studying and monitoring its distribution is costly. Currently, many opportunistic sightings (OS) reports are available, which provide a source of potentially useful, low-cost information about the spatio-temporal distribution of this species. Since 1993, the Spanish Institute of Oceanography has compiled a dataset comprising 874 records of OS of nine species of cetaceans in the western Mediterranean Sea and adjacent waters. The aim of this study was to use this dataset to investigate the differential use of these waters by the fin whale when compared with other cetaceans. We compared the presence of fin whales with the presence of any other cetacean species in the dataset. Binary logistic regression was then used to model these occurrences according to several spatio-temporal variables expected to reflect their habitat use. Several significant models reveal that fin whales are more prone than other cetaceans to use the waters over the slope of the Gulf of Lion in summer. This finding confirms that the Gulf of Lion is an area of importance for this species and suggests that the slope of the continental shelf could be particularly important. Our study shows how OS can be a source of useful information when appropriately analyzed.

## Introduction

The Mediterranean Sea is home to 11 regular cetacean species (Notarbartolo Di Sciara et al., 2016): seven species from the family Delphinidae, and one species each from the Balaenopteridae, Physeteridae, Phocoenidae and Ziphiidae. The Balaenopteridae species is the fin whale (*Balaenoptera physalus*, Linnaeus, 1758). The general pattern of fin whale migration is to move annually from high latitudes in summer to lower latitudes in winter, where they find appropriate foraging and breeding grounds, respectively (Corkeron & Connor, 1999). These migratory movements suggest that the fin whale could have spatio-temporal patterns in habitat use different from those of other cetaceans in the Mediterranean, although this has still to be explored in the latitudinally limited Mediterranean Sea.

The fin whale is globally classified as “endangered” by the International Union for Conservation of Nature (IUCN) Red List of Threatened Species, although the North Atlantic population is increasing (Reilly et al., 2013). There are fin whales in the Mediterranean Sea that are genetically different from those inhabiting the North Atlantic Ocean (Bérubé et al., 1998; Palsbøll et al., 2004). Fewer than 5,000 fin whales are assumed to occur in the whole Mediterranean basin, where the species is known to be declining and is classified as Vulnerable by the IUCN Red List of Threatened Species (Panigada & Notarbartolo Di Sciara, 2012). Fin whales are of conservation concern due to cumulative natural and anthropogenic threats in this semi-enclosed basin (Thomas, Reeves & Brownell, 2015). These threats include disease outbreaks (Mazzariol et al., 2016), ship strikes (Panigada et al., 2006), plastic ingestion (Fossi et al., 2016) and underwater noise (Castellote, Clark & Lammers, 2012).

Bentaleb et al. (2011) suggested that most of the fin whales remain in the western Mediterranean Sea the whole year. Although there are no Mediterranean-wide estimates of fin whale abundances, such estimates are available for certain areas and seasons that received line-transect survey coverage. Recent aerial surveys conducted in the north-western Mediterranean, off the coasts of France and Italy, provided an estimate of 2,500 fin whales in summer and 1,000 fin whales in winter, which suggests a seasonal trend in the area (Laran et al., 2017). The lower abundance in the northwest Mediterranean winter may be due to fin whale movements from the north-western Mediterranean to the Lampedusa Island (Canese et al., 2006; Aïssi et al., 2008) and the Alboran Sea (Cotté et al., 2011). These movements affect also individuals coming from the North-Atlantic, which tend to go to the north-western Mediterranean Sea (Giménez et al., 2013). Gauffier et al. (2018) suggested that this migration through the Strait of Gibraltar is bidirectional with a seasonal pattern toward the Atlantic Ocean mainly between May and October and toward the Mediterranean Sea mainly between November and April. Nevertheless, Geijer, Notarbartolo Di Sciara & Panigada (2016) suggested that the highly dynamic migratory behavior of resident Mediterranean fin whales is not yet completely understood. Consequently, greater knowledge of ecological and biogeographical factors affecting fin whales is needed to identify their specific habitat use in the Mediterranean (Castellote, Clark & Lammers, 2011).

In general, research in the marine environment needs the use of a floating or airborne vehicle, usually leading to higher costs than terrestrial research. Monitoring fin whale distribution is particularly expensive because this species typically makes long migrations on the high seas, distant from main ports and airfields, and shipboard or aerial surveys must cover vast areas. Several studies of the distribution of cetacean species have reduced costs by using platforms of opportunity such as ferries, which record the associated search effort (Kiszka et al., 2007; Moura, Sillero & Rodrigues, 2012; Aïssi et al., 2015; Cominelli et al., 2016). In order to further reduce research costs, several authors have proposed the use of opportunistic sightings (OS) using different information sources not limited to fixed routes (Siebert et al., 2006) but with no control of sampling effort (but see Himes Boor & Small, 2012). This type of data provides relevant information at relatively low cost (MacLeod, Brereton & Martin, 2008; Moura, Sillero & Rodrigues, 2012; Aïssi et al., 2015).

The fin whale is easily identified due to its large size, characteristic blow, and fusiform shape (Cominelli et al., 2016) and is the only mysticete species that seems to be resident in the Mediterranean Sea (IUCN, 2012). This makes OS presence data for this species in the Mediterranean highly reliable. However, the detection and observation biases inherent to this kind of non-dedicated survey makes absence data less reliable. The detection of cetaceans is affected by an availability bias, when observers fail to detect animals because they are not available, that is, submerged, and a perception bias, when observers fail to detect potentially visible animals, that is, present at the surface (Marsh & Sinclair, 1989). More importantly, opportunistic observations are not planned in advance, and a lack of presence may be due to a lack of observation, resulting in an unknown bias. Consequently, OS data likely represent a biased fraction of the true distribution of the species (McDonald, Mesnick & Hildebrand, 2006; Praca et al., 2009; McClellan et al., 2014). This precludes the use of these data to model the overall distribution of the species. However, OS data allow comparing the differential distribution of fin whales with respect to that of other species from the same dataset, as the biases and the reliability of both kind of data are essentially the same.

The Spanish Institute of Oceanography (Spanish acronym; IEO) has collected OS data in recent decades, with more than 1,000 observations of marine megafauna, mostly cetaceans.

The aim of this study was to use the OS data collected by the IEO to identify the differential spatio-temporal use of the western Mediterranean Sea and adjacent waters by fin whales compared to other cetaceans, and to consider the implications for conservation management of this species.

## Material and Methods

### Study area and data collection

The study area comprised the western Mediterranean Sea and adjacent waters (35°–43°N; 9°W–15.5°E), an area known to be frequented by cetaceans. This area includes several Important Marine Mammal Areas (IMMAs) as identified by the IUCN (Fig. S1).

From 1993 to 2014, the IEO has compiled a database comprising 874 records of OS of nine species of cetaceans (Tables S1 and S2). This database included 70 OS of fin whales. Most of the data were collected by IEO staff and trained scientific fishery observers. Cetacean identification forms were also given to volunteers and frequent users of the sea (e.g., sailboat users) interested in collaborating with the IEO, who contributed with 9% of the available OS data and 8.6% of data about fin whales. Fin whale sightings were not recorded every year (Table S1).

Opportunistic sighting data included position, time, date, type of vessel and observer identification, and sometimes included bearing, size of group, and sea state. Each entry represents an OS of a single cetacean or a group that could include one or several species. In some cases, fin whales and another cetacean species were sighted together, and we separated these cases into two different entries, namely the sighting of fin whale and the sighting of other cetacean. We classified the data as OS of the fin whale and OS of other species combined, and this was the binary dependent variable which was modeled.

### Explanatory variables

We used the time and location of each sighting to derive several spatio-temporal variables, which were selected on the basis of their expected capacity to describe the large-scale patterns influencing the distribution of the fin whale. These variables could be related with environmental, geographic or anthropogenic factors that may differentially affect this species (Cañadas, Sagarminaga & García-Tiscar, 2002; Kiszka et al., 2007; Aïssi et al., 2008; Laran & Gannier, 2008; Schipper et al., 2008; Panigada et al., 2011; Rako et al., 2013). All the distances were measured as the shortest distance from each OS to an object with the “Near” tool of ArcGIS 10.1 software (ESRI Inc., Redlands, CA, USA) in kilometers with a precision of 0.5 km.

**Figure 1:**
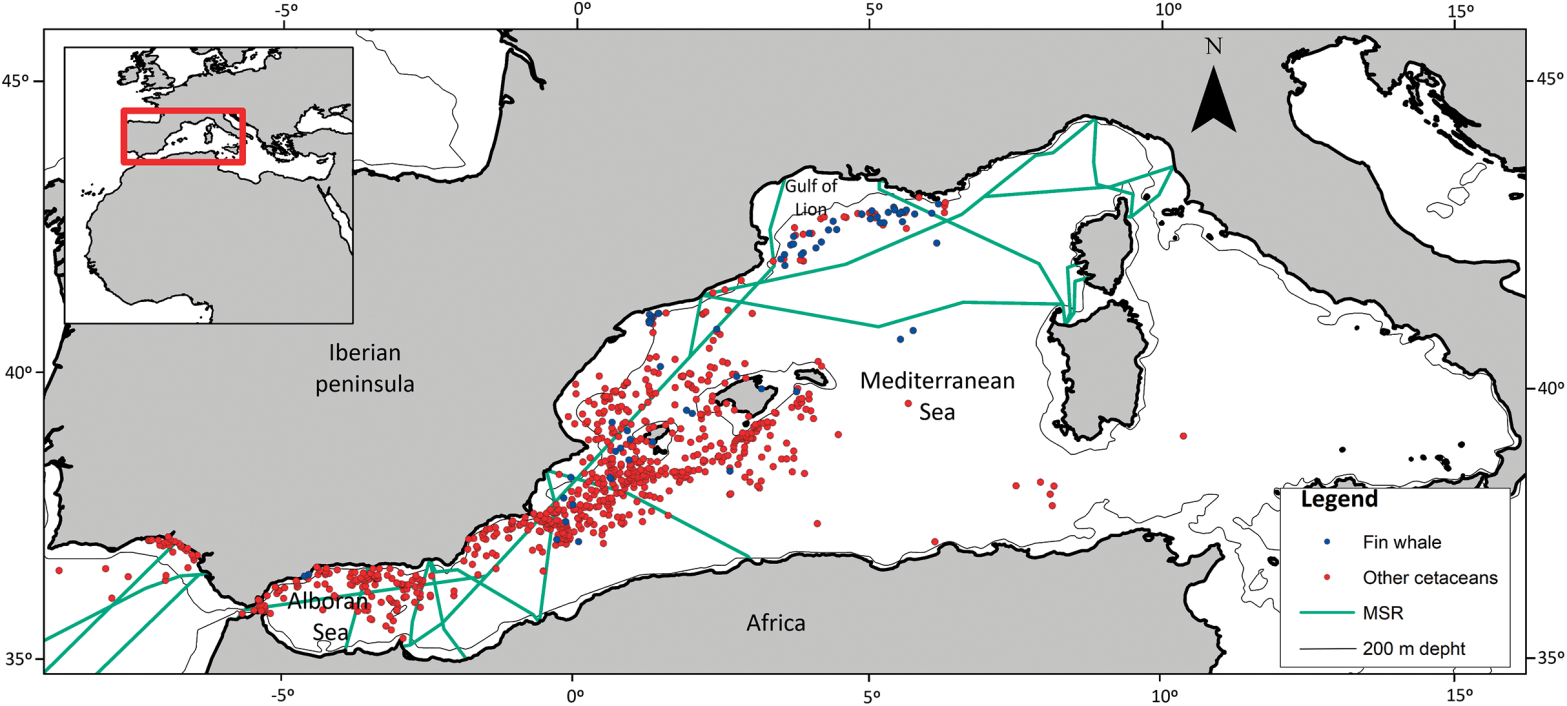
Spatial location of cetacean opportunistic sightings. Study area, location of opportunistic sighting data of fin whales and of the other cetacean species combined, isobaths of 200 m, and marine shipping routes. Blue dots, fin whales; red dots, other cetaceans. MSR, Marine Shipping Routes.

We used the following spatial variables:

Continental Shelf (CS): CS is a binary and dimensionless variable, on or off the CS (Fig. 1) created for each OS. The CS was taken to be less than the 200 m depth isobath (available at: http://www.naturalearthdata.com/downloads/10m-physical-vectors/10m-bathymetry). ArcGIS 10.1 software was used to establish if the OS occurred on or off the CS.

Distance to coast (DC): DC is a continuous variable that was used to measure the effect of coastal areas on the marine ecosystems (Aïssi et al., 2014). In general, as distance increases, the influence of the continent decreases and the predominance of the pelagic ecosystem increases. Values for this variable were obtained using the coastline layer (available at: http://www.naturalearthdata.com/downloads/10m-physical-vectors/10m-coastline/). These values ranged from zero to 172 km.

Geographical trend: Population dynamic processes, such as natality, mortality or migration, influence the distribution of species apart from the influence of the environment. This results in a purely spatial structuring in the distribution of observations that is functional rather than spurious and noise-producing, and which should be included in species distribution modeling (Legendre, 1993). As each OS was recorded by latitude (La) and longitude (Lo) in degrees, these geographical coordinates were introduced in the models as explanatory variables to test for the existence of differential spatial trends in the form of latitudinal or longitudinal gradients in the distribution of fin whale sightings (Druon et al., 2012; Cominelli et al., 2016).

Distance to main shipping route (MSR): MSR is a continuous variable that was used to assess whether large commercial vessels differentially affect the spatial pattern of fin whale sightings (Fig. 1), as a result of disruptive underwater noise or fatal collisions (Castellote, Clark & Lammers, 2012; Panigada et al., 2006). Google Maps (available at: https://www.google.es/maps/) was used to generate a layer of the MSRs within the research area. The shortest distance in km from the OS to the main route was calculated, with values ranging from zero to 824 km.

Distance to coastal cities with more than 100,000 inhabitants (CiD): CiD is a continuous variable that was used to test whether cities influenced the probability of OS. This variable is a proxy of the negative anthropogenic effect in nearby areas given that cities are pollution sources. A layer was created of cities of more than 100,000 inhabitants in the study area, using online information (such as the National Statistics Institute) to check the population size of the cities. Distances ranged from two to 268 km.

We also used the following temporal variables:

Moon phase (MP): MP is a categorical variable that was used to determine if the temporal pattern of tides had any effect on the probability of sighting. This variable has been studied in other pelagic species (Dos Santos & Garcia, 2005; Poisson et al., 2010). The phases used were: new moon, first quarter, full moon, and third quarter.

Season: Given the migratory behavior of this species, we tested potential seasonal effects on the distribution of sightings (Druon et al., 2012). This categorical explanatory variable may take any of four states: spring (Sp, March 21–June 20); summer (Su, June 21–September 22); autumn (Au, September 23–December 21); and winter (Wi, December 22–March 20).

### Model building

In the present study we used binary logistic regression (Hosmer & Lemeshow, 2000), using two kinds of presence data as the two states of the binary variable to be modeled, by comparing the OS of the fin whale with the OS of other species combined (Romero & Real, 1996; Arntzen & Espregueira, 2008). Logistic regression models are commonly used to investigate cetacean-habitat relationships, normally using presence-absence data that require recording the observed absences, which is lacking here (Redfern et al., 2006). However, if the presence of a cetacean species is compared with the presence of other cetacean species in the same dataset, then the sampling effort and bias may be considered to be the same for the two data subsets under comparison, and the resulting differential patterns are not attributable to the lack of a dedicated sampling. However, as no information is included in OS datasets about observation of absence of any cetacean species, the resulting distribution models should be interpreted in terms of the differential distribution of the fin whale with respect to that of other species of the same order (Niamir et al., 2016).

When several explanatory factors are interrelated, as is the case here, it is common that the effects of some of them are obscured or inflated by those of others (Cartron, Kelly & Brown, 2000; Real et al., 2013). This is reflected in type I/II statistical errors that affect each individual test. Consequently, the true effect of each factor should be assessed in the context of the other evaluated influences. On the other hand, multivariate techniques are capable of revealing complex relations between environmental factors, but sometimes make some questionable assumptions that render their results doubtful of biological validity (James & McCulloch, 1990). Because of this, we first created univariate explanatory logistic regression models to assess each of the eight spatio-temporal variables individually. We also performed a forward–backward stepwise logistic regression on all the variables to investigate whether a combination of spatio-temporal factors could better account for the differential distribution of the species than univariate models. However, all statistically significant results may provide meaningful information. We discuss all significant results, both univariate and multivariate, taking into account the interrelation between the different evaluated factors. This is in accordance with Hosmer & Lemeshow (2000, pp. 92–99) recommendation for model-building strategies for logistic regression.

The statistical significance of the models was established according to the Omnibus test (Legendre & Legendre, 1998), and the Wald test was used to test the statistical significance of the individual regression coefficients introduced in the multivariate model (Peng, Lee & Ingersoll, 2002). All significant models were thoroughly evaluated (see below), ranked and discussed, as all of them represent a use of space and time by fin whales more different from those made by other cetaceans than expected at random, and thus provide meaningful information. We used the software IBM SPSS Statistics 25 (IBM, Armonk, NY, USA) to perform all the statistical analyses described above.

### Model evaluation

We evaluated the parsimony, calibration, and discrimination capacity of each significant model. All the models were ranked according to their parsimony measured by the Akaike Information Criterion (AIC) (Akaike, 1973). The calibration of the models was determined using the Hosmer & Lemeshow test, which compares observed and expected frequencies of the binomial variable for each range of the probability value and assesses the general adjustment of the model (Hosmer & Lemeshow, 2000). In a well-calibrated model, there are no significant differences between the observed and expected frequency distributions (Peng, Lee & Ingersoll, 2002). The discrimination capacity of the models was assessed by the area under the receiver operating characteristic curve, known as the area under the curve (AUC) (Lobo, Jiménez-Valverde & Real, 2008), which gives the probability that a random sighting of fin whale has a higher model output value than a random sighting of other cetacean.

## Results

Figure 1 shows the spatial distribution of the 70 sightings of fin whales and the 804 sightings of other cetaceans. There were few OS of fin whales in the Atlantic coast, Strait of Gibraltar and Alboran Sea, whereas they were more frequent in the Gulf of Lion area. Fin whale observations occurred in all seasons, indicating that they are present in the western Mediterranean Sea all year long. Forty-four OS (nearly 63%) were in the North West Mediterranean Sea, Slope, and Canyon System IMMA (IUCN-MMPATF, 2017) (Fig. 1).

We obtained three significant univariate models, two of them spatial, including La and Lo, respectively, and one temporal model including season (Table 1). Non-significant models are also listed in Table 1. The model with La as an explanatory variable was the best univariate model according to the AIC (Table 1, Model 2), indicating that the probability that a sighting corresponded to fin whale was higher in the northern areas. A significant positive relationship was also found between longitude Lo (Table 1, Model 3) and fin whale OS, with the probability of sighting a fin whale being higher in the eastern areas. The temporal model (Table 1, Model 4) showed that the sighting of a fin whale was relatively more probable compared with the probability of sighting other cetacean species in summer and less probable in autumn. This indicates that the fin whale makes differential use of these waters in summer, when most OS of fin whale occurred, and quits these waters in autumn more than other cetacean species do.

**Table 1:** Significant and non-significant models and their evaluation measures.

Model	Variables	Logit	Omnibus test	AIC^1^	AUC^2^	Hosmer & Lemeshow test
Significant models						
1	La + CS	$ - 32.170 + 0.771 \times {\rm{La}} + \left\{ {\matrix{{{\rm{0\;if\;sighting\;is\;on\;the\;CS}}} \cr { - {\rm{0}}{\rm{.803\;beyond\;the\;CS}}}\cr } } \right.$	χ^2^ = 131.339; d.f. = 2; *P* < 0.001	362.340	0.845	χ^2^ = 6.107; d.f. = 8; *P* = 0.635
2	La	−31.975 + 0.750 × La	χ^2^ = 126.607; d.f. = 1; *P* < 0.001	365.073	0.841	χ^2^ = 4.213; d.f. = 8; *P* = 0.837
3	Lo	−2.952 + 0.347 × Lo	χ^2^ = 59.360; d.f. = 1; *P* < 0.001	432.320	0.782	χ^2^ = 19.012; d.f. = 8; *P* = 0.015
4	Season	$ - 2.539 + \left\{ {\matrix{ { - 0.214{\rm{\,Sp}}} \cr {0.433{\rm{\,Su}}} \cr { - 1.268{\rm{\,Au}}} \cr {0{\rm{\,Wi}}} \cr } } \right.$	χ^2^ = 11.998; d.f. = 3; *P* = 0.007	479.682	0.607	χ^2^ = 0.000; d.f. = 2; *P* = 1.000
Non-significant models						
5	Moon phase	$ - 2.220 - \left\{ {\matrix{ {{\rm{0}}{\rm{.525\,new\,moon}}} \cr {{\rm{0}}{\rm{.093\,first\,quarter}}} \cr {{\rm{0}}{\rm{.965\,full\,moon}}} \cr {{\rm{0\,third\,quarter}}} \cr } } \right.$	χ^2^ = 6.315; d.f. = 3; *P* = 0.097	483.365	0.575	χ^2^ = 0.000; d.f. = 2; *P* = 1.000
6	CS	$ - 2.097 + \left\{ {\matrix{ {{\rm{0\;if\;sighting\;is\;on\;the\;CS}}} \cr { - {\rm{0}}{\rm{.414\;beyond\;the\;CS}}} \cr } } \right.$	χ^2^ = 1.610; d.f. = 1; *P* = 0.205	488.070	0.529	Non applicable (d.f. = 0)
7	CiD	−2.163 − 0.003 × CiD	χ^2^ = 1.353; d.f. = 1; *P* = 0.245	488.326	0.552	χ^2^ = 14.097; d.f. = 8; *P* = 0.079
8	MSR	−2.332 + 0.002 × MSR	χ^2^ = 0.990; d.f. = 1; *P* = 0.320	488.689	0.456	χ^2^ = 20.764; d.f. = 8; *P* = 0.008
9	DC	−2.615 + 0.004 × DC	χ^2^ = 0.790; d.f. = 1; *P* = 0.374	488.890	0.520	χ^2^ = 9.281; d.f. = 8; *P* = 0.319

**Notes:**

Significant models describing the differential spatio-temporal use of the study area by the fin whale in relation to other cetacean species, ranked according to AIC values, and non-significant models. The logit functions derived from logistic regression of a binary variable (sighting of fin whale against sighting of other cetacean) on the spatio-temporal variables. The Omnibus test gives the statistical significance of the models, AIC evaluates their parsimony, AUC assesses their discrimination power, and Hosmer & Lemeshow test evaluates their calibration.

La, Latitude; Sp, Spring; Su, Summer, Au, Autumn; Wi, Winter; Lo, Longitude; CS, Continental shelf; CiD, Distance to coastal cities with more than 100,000 inhabitants; MSR, Distance to main shipping route; DC, Distance to coast; χ^2^, Chi-squared distribution; d.f., degrees of freedom; *P*, probability value.

1 Akaike Information Criterion.

2 Area under the receiver operating characteristic (ROC) curve.

**Figure 2:**
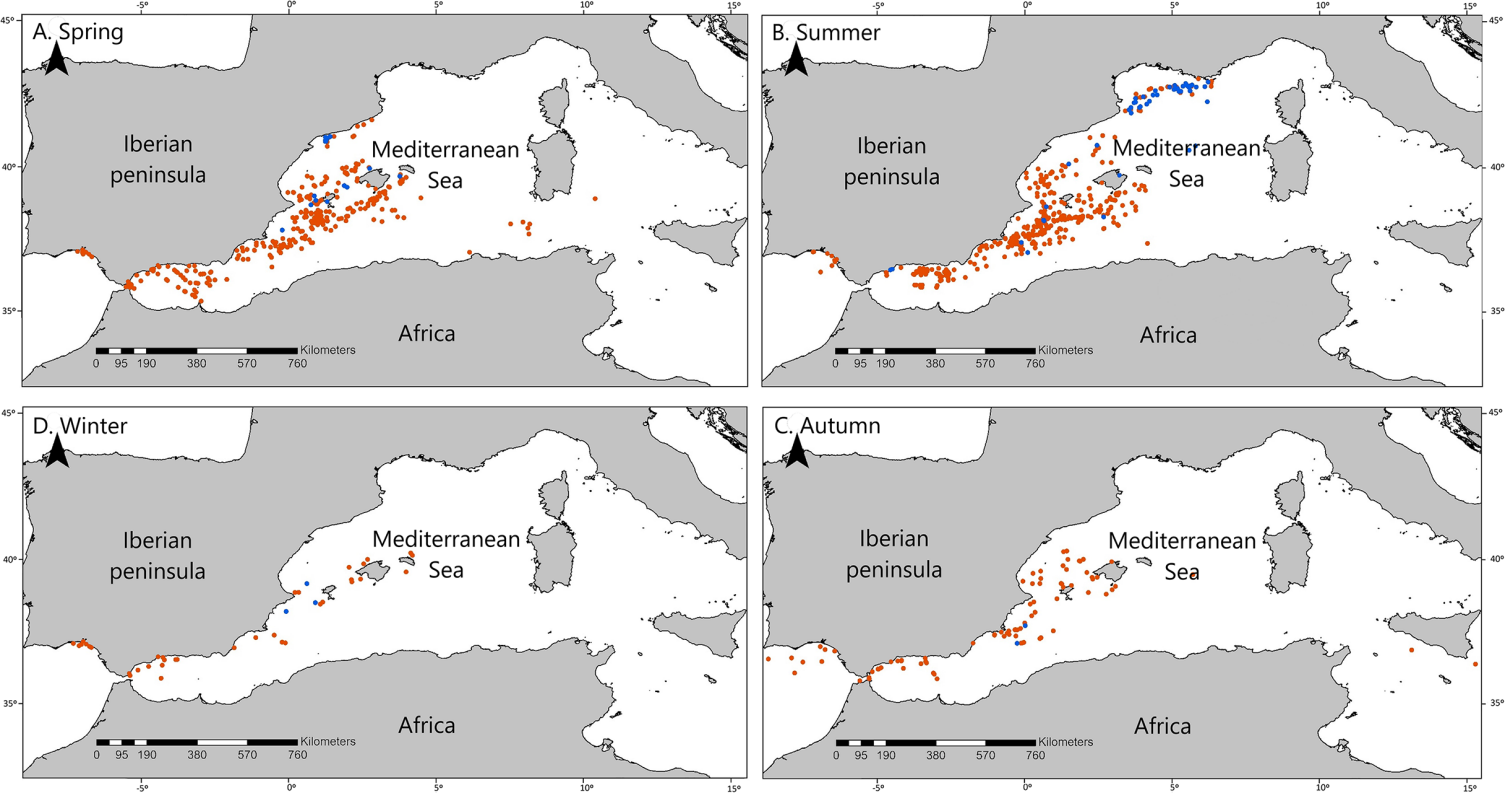
Seasonal distribution of the opportunistic sighting dataset of cetaceans. Cetacean opportunistic sightings in each season. (A) Spring, (B) Summer, (C) Autumn, and (D) Winter. All maps show fin whale sightings in blue and non-fin whale cetaceans in red.

**Figure 3:**
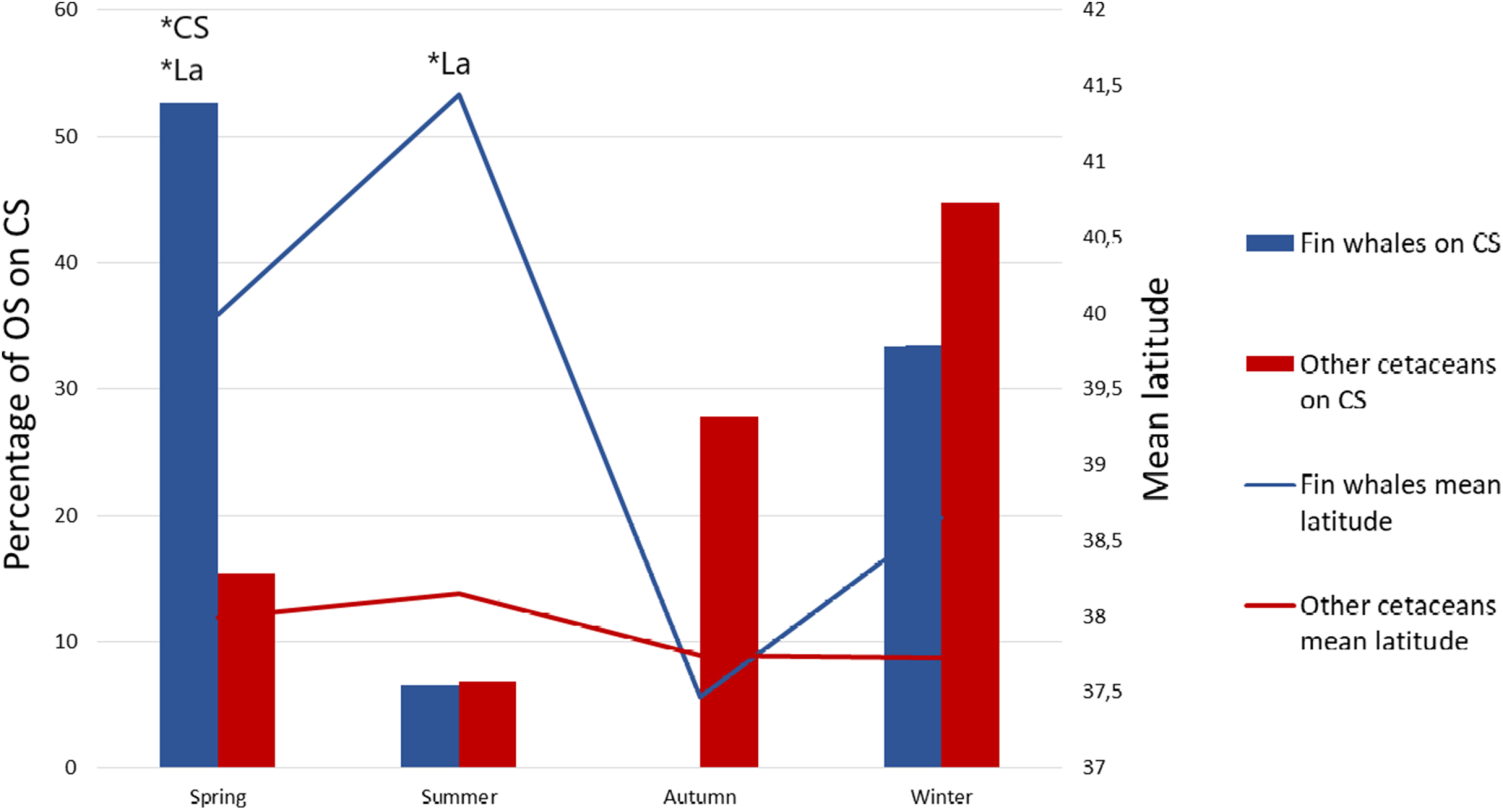
Mean latitude and percentage of sightings on the continental shelf as a function of season. Percentage of opportunistic sightings (OS) on the continental shelf (CS) for the fin whale (blue bars) and for the other cetacean species pooled (red bars) during each season, and mean latitude of the OS of fin whales (blue line) and of the other species pooled (red line) per season. *P < 0.01 in the Omnibus test performed on separate logistic regression models of OS data on La and CS for each season. In all non-significant models the significance value was *P* > 0.05.

We also obtained one significant multivariate model including La and CS, which indicates that fin whales are differentially found in northern latitudes and on the CS (Table 1, Model 1). The best model, according to AIC values, was this multivariate model (Table 1). Latitude was more significant than CS according to Wald test values (Wald = 104.366, *P* < 0.001 for La, Wald = 5.13, *P* = 0.024 for CS). This means that the probability that an OS corresponds to fin whales is higher in the north and, secondarily, the probability is higher on the CS. However, the effects of La and CS on OS of fin whales were interrelated with those of season, as the northernmost OS of fin whales occurred in summer and the OS of fin whale on the CS occurred mainly in spring (Table 2; Figs. 2 and 3). To assess possible interactions between these variables and season, we built separate models of La and CS for each season. La was only significant during the summer season, when mean latitude for fin whale OS was 3° higher than that of OS of other species, and to a lesser extent during springtime (Fig. 3). CS was only significant during spring (Table 2; Fig. 3). This is the reason why season was not included in the multivariate model, since the effects of La and CS incorporate those of season.

**Table 2:** Seasonal distribution of the opportunistic sighting dataset, and its relationship with continental shelf.

Season	OS		On continental shelf	
	Fin whales	Other cetaceans	Fin whales	Other cetaceans
Spring	19	298	10	46
Summer	46	378	3	26
Autumn	2	90	0	25
Winter	3	38	1	17
Total	70	804	14	114

**Note:**

Frequency of opportunistic sightings (OS) of fin whales and other cetaceans by season and their corresponding frequency on the continental shelf.

## Discussion

In general, our results should be interpreted as referring to a group of spatial and temporal variables that could explain the differential distribution of fin whales from a macroecological perspective (Niamir et al., 2016). The models do not predict the probability of a fin whale sighting; rather, they explain the differential use of habitat by fin whales in relation to the other species of cetaceans that were sighted.

### The importance of the Gulf of Lion

Our results show that fin whales were differentially sighted in the north–east part of the western Mediterranean basin, on the CS and in summer (Tables 1 and 2; Fig. 2). In fact, 47% of fin whale sightings (33 out of 70) occurred in the Gulf of Lion, at the northern limit, and to a lesser extent the eastern limit, of the study area (Fig. 1). This suggests that the effects of latitude and longitude point to the Gulf of Lion, which is an important area for fin whales. As all these 33 sightings occurred in summer, we may conclude that the Gulf of Lion is important for fin whales in summer.

The Gulf of Lion is close to the Pelagos Sanctuary, which is a known important area for the fin whale (Cotté et al., 2009; UNEP-MAP-RAC/SPA, 2013; Cominelli et al., 2016). There is an international agreement between France, Monaco, and Italy for the protection of all marine mammals in the Pelagos Sanctuary (Notarbartolo Di Sciara et al., 2008; UNEP-MAP-RAC/SPA, 2013), as it supports a high diversity of cetaceans (Aïssi et al., 2015), but this protection does not cover the Gulf of Lion. The Gulf of Lion has been previously identified as an important area for fin whales where they tend to concentrate (Cotté et al., 2009; Bauer et al., 2015; Notarbartolo Di Sciara et al., 2016; Laran et al., 2017; Lambert et al., 2017). The North West Mediterranean Sea, Slope, and Canyon System IMMA (IUCN-MMPATF, 2017) includes the Gulf of Lion, and almost 63% of our fin whale OS were situated in that important area for marine mammals (Fig. S1). In addition, these sightings particularly concentrated on the slope. We confirm that this area is indeed important for the fin whale, and suggest that the continental slope of the Gulf of Lion should be also granted the same level of protection conferred to the Pelagos Sanctuary.

Druon et al. (2012) used a dataset only with the presences of fin whales which included many of sightings in the western Liguro-Provençal Basin, an area which encompass the Pelagos Sanctuary and the Gulf of Lion. They reported few fin whales sightings over the continental slope of the Gulf of Lion, but as they did not report sampling coverage, it is uncertain whether this was due to lack of observation effort or to true absence.

Our results also confirm that the presence of fin whales is much lower outside the Liguro-Provençal Basin. This may explain why, for example, Boisseau et al. (2010), who conducted a survey of the Mediterranean Sea and adjacent waters (excluding the Liguro-Provençal basin), did not identify any fin whales. However, our study did not identify other known important areas such as the Alboran Sea (Castellote, Clark & Lammers, 2012; Gauffier et al., 2018).

### The effect of the Continental Shelf

Our multivariate model revealed that CS was a relevant explanatory variable, although according to our univariate models it was not significantly related to the differential distribution of the fin whale. The effect of the CS was initially obscured by the related effect of La, because in the Gulf of Lion the fin whales were not observed on the CS, but when La was included in a stepwise multivariate model, the effect of CS became significant (*P* = 0.021), as it explained the residual effects that were not explained by La alone.

Fin whales are thought to be mainly pelagic (Notarbartolo Di Sciara et al., 2003; Aïssi et al., 2008; Bauer et al., 2015), although they are also found on the shelf and outer slope (Panigada et al., 2005; Azzellino et al., 2008; Druon et al., 2012). Our data is in line with these findings because 56 out of the 70 fin whale sightings were observed off the CS, and 33 of these sightings occurred on the slope of the Gulf of Lion. Prey availability, influenced by bathymetry and oceanographic processes, is known to be a driving force for the distribution of this species (Canese et al., 2006; Aïssi et al., 2008; Cotté et al., 2009, 2011), and this could be the cause of the differential concentration of fin whale sightings on the slope of the Gulf of Lion. However, our results suggest that in spring, when more than half of fin whale sightings occurred on the CS, fin whales could be mainly migrating to the Gulf of Lion following more shallow coastal routes (Fig. 2). Canese et al. (2006) described fin whales feeding on *Nyctiphanes couchii* (Bell, 1853) in winter in shallow waters of the area of Lampedusa. We recommend performing a similar study in the waters of the eastern Iberian Peninsula during the spring, which could help to describe new feeding grounds for fin whales in the Mediterranean Sea, particularly in the context of the Cetacean Migration Corridor in the western Mediterranean Sea (Real Decreto 699/2018, 29 June, Ministerio para la Transición Ecológica).

### The seasonal pattern

The effect of season, which was apparent in the univariate models, remained obscured by those of La and CS in the multivariate model, because in summer the OS of fin whales occurred mainly in the north, whereas in spring occurred mainly on the CS, and those variables were already included in the model, which made season redundant. In summary, the three spatio-temporal variables, namely La (which points to the Gulf of Lion), CS and season, are relevant and interrelated, and reflect the differential spatio-temporal use of these waters by fin whales when compared with other cetaceans.

The seasonal behavior of the resident Mediterranean fin whales is highly dynamic, and there is no generally accepted seasonal pattern for this species (Geijer, Notarbartolo Di Sciara & Panigada, 2016). Panigada et al. (2011) found that sightings of fin whales were more common in the Pelagos Sanctuary in summer. Our results concur with this finding, as the Gulf of Lion, which is close to the Pelagos Sanctuary, is more favorable for fin whales than for other cetaceans in summer. Aïssi et al. (2008), detected more sightings of fin whales in the Pelagos Sanctuary in summer, whereas autumn and winter sightings concentrated in the Strait of Mesina and in the area of Lampedusa until the beginning of spring. This may explain why OS of fin whales were differentially scarce in autumn in our study area (Table 2; Fig. 2). These results suggest that there is spatial and temporal variability within the central Mediterranean Sea (Aïssi et al., 2008), which coincides with a migration pattern toward higher latitude within the western Mediterranean Sea in summer, which has been previously identified (Panigada et al., 2006; Bentaleb et al., 2011; Castellote, Clark & Lammers, 2012; Druon et al., 2012; Cotté et al., 2009; Arcangeli, Campana & Bologna, 2017). Moreover, some authors showed that fin whales exhibited fidelity to the northwestern Mediterranean Sea with a summer-aggregated and winter-dispersed pattern (Cotté et al., 2009). Mediterranean fin whales seem to feed mainly on krill *Meganyctiphanes norvegica* (M. Sars, 1857), which concentrate in the Gulf of Lion in summer and could be a driving force for the seasonal aggregation behavior described here (Bentaleb et al., 2011).

### The effect of distance to main shipping routes

None of the significant models included the variable distance to MSRs. However, fin whales are at risk of ship strikes. They also appear to be one of the species in the Mediterranean Sea most affected by underwater noise, as they seem to change their behavior in response to this noise or flee from underwater sound sources (Castellote, Clark & Lammers, 2012; Sciacca et al., 2015). The variable MSR was used to assess the differential influence of noise and ship strikes on fin whale distribution in comparison with other cetaceans, and the fact that we did not find any significant relationship with this variable does not mean that they do not affect fin whales (Fig. 1). This only means that shipping routes affect fin whales in a similar way as they affect other cetacean species, so that no differential effect was found in our analysis.

### How can we get conclusions from opportunistic sightings?

The principal methods used for the study of cetacean distribution and abundance are line transect sampling from vessels or aerial surveys (Cañadas & Hammond, 2006; Báez, Camiñas & Torreblanca, 2007; Gómez De Segura, Hammond & Raga, 2008; Panigada et al., 2011) and photo-identification (Cañadas & Sagarminaga, 2000; Auger-Méthé & Whitehead, 2007; Carpinelli et al., 2014). These studies make it possible to estimate species abundance and the variables that determine their distribution at small or intermediate scales. The information provided by these approaches could be useful when attempting to delimit protected marine areas. Nevertheless, these scientific methods are costly and require survey vessels or airplanes.

The main advantage of OS approaches based on platforms of opportunity is their low cost, since they take advantage of various marine trips to gather sightings information. Although such datasets may include some unreliable sightings, the issue of reliability is of less concern in our case, given the participation of experienced specialists in the OS and the ease of identification of the fin whale. The method used here with this OS dataset provided significant models that identified certain factors affecting the differential distribution of this species, which is especially relevant when there is a lack of dedicated surveys (Pikesley et al., 2012). The limitation of this method is that, due to the lack of a dedicated sampling, the interpretation of the results should be limited to the differential spatio-temporal use of the study area by the target species when compared with the other species in the same dataset. Specifically, we used a dataset with only presences in a similar way as other studies such as Esteban et al. (2013) and Sánchez-Cabanes et al. (2017), which provide useful information about the differential distribution of the analyzed cetacean species. This information is valuable when attempting to infer key areas for the life cycle of the species, as is the slope of the Gulf of Lion for fin whales in summer.

## Conclusions

We conclude that the fin whale has a differential spatio-temporal distribution when compared with other cetaceans in the western Mediterranean Sea. In summer, fin whales are differentially concentrated in the slope off the CS in the Gulf of Lion, which reinforces the evidence about the importance of this area for the fin whale. A novelty of our approach lies in the utilization of OS to draw conclusions on the species differential habitat use. OS contributed to understand the differential spatio-temporal distribution of the fin whale in the western Mediterranean and helped corroborate factors previously identified in other studies. In a similar way, the OS could provide useful information about the differential spatial use of other endangered marine species for which more systematic data are scarce.

## Supplemental Information

10.7717/peerj.6673/supp-1 Supplemental Information 1 Opportunistic sightings of cetaceans used to create the models.

10.7717/peerj.6673/supp-2 Supplemental Information 2 Cetacean species.

10.7717/peerj.6673/supp-3 Supplemental Information 3 Location of Important Marine Mammal Areas according to the IUCN, and of the opportunistic sightings.

## Additional Information and Declarations

### Data Availability

The following information was supplied regarding data availability: The raw data are available in the Supplemental Files.
