# Supplementary figures and images for: Using opportunistic sightings to infer differential spatio-temporal use of western Mediterranean waters by the fin whale
Source: PeerJ. 2019 Mar 29;7:e6673. doi: 10.7717/peerj.6673 (PMC6442671; doi:10.7717/peerj.6673)

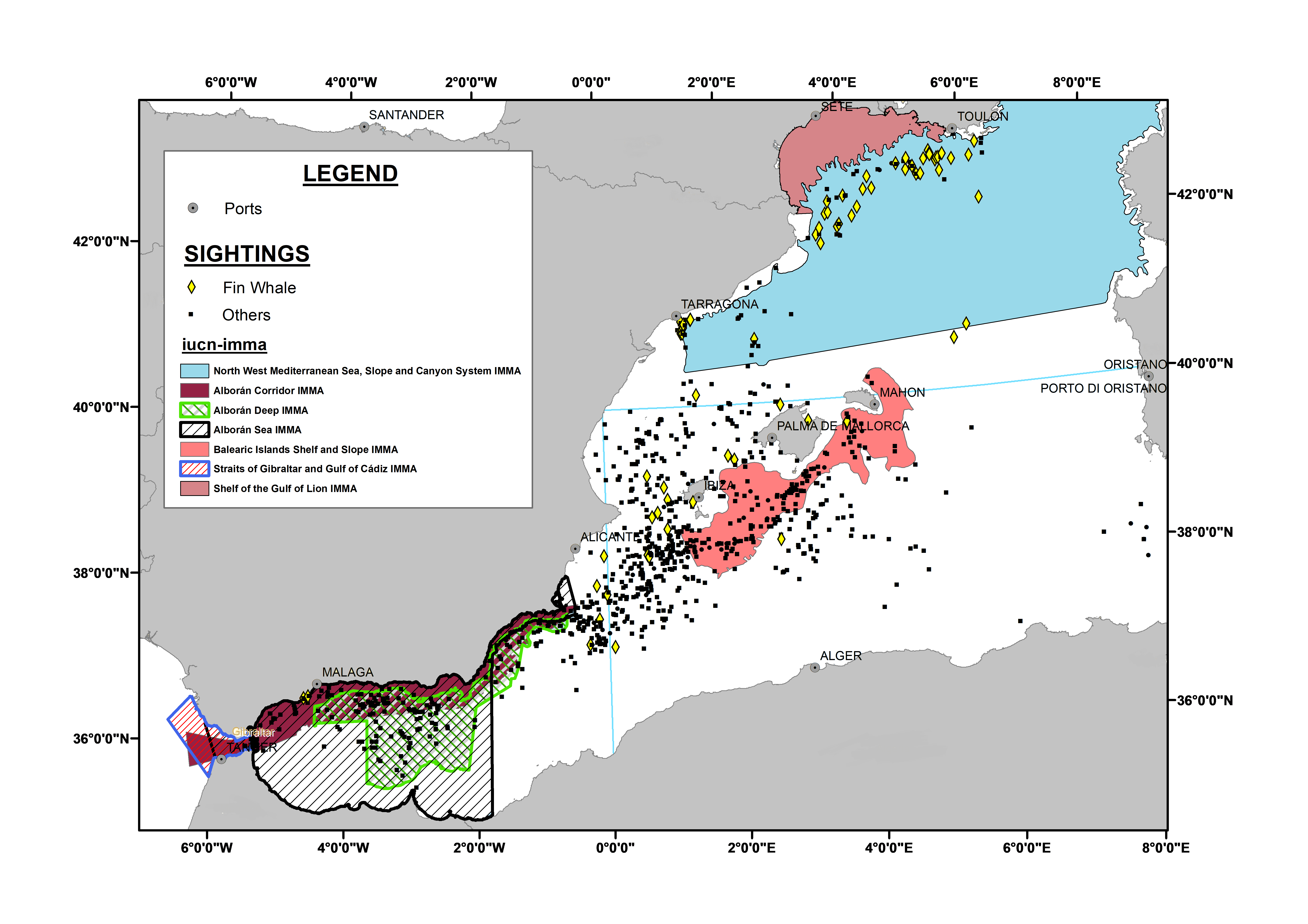

Supplement: Supplemental Information 3 [file peerj-07-6673-s003.png]
